# Response of stomatal density and size in *Betula ermanii* to contrasting climate conditions: The contributions of genetic and environmental factors

**DOI:** 10.1002/ece3.11349

**Published:** 2024-06-18

**Authors:** Yihan Cai, Takaki Aihara, Kyoko Araki, Ragini Sarmah, Yoshihiko Tsumura, Mitsuru Hirota

**Affiliations:** ^1^ Graduate School of Environmental Science Hokkaido University Nayoro Japan; ^2^ Graduate School of Life and Environmental Sciences University of Tsukuba Tsukuba Japan; ^3^ Graduate School of Science and Technology University of Tsukuba Tsukuba Japan; ^4^ Faculty of Life and Environmental Sciences University of Tsukuba Tsukuba Japan

**Keywords:** *Betula ermanii*, climate change, common garden experiment, intraspecific variation, stomatal density, stomatal size

## Abstract

As plant distribution and performance are determined by both environmental and genetic factors, clarifying the contribution of these two factors is a key for understanding plant adaptation and predicting their distribution under ongoing global warming. *Betula ermanii* is an ideal species for such research because of its wide distribution across diverse environments. Stomatal density and size are crucial traits that plants undergo changes in to adapt to different environments as these traits directly influence plant photosynthesis and transpiration. In this study, we conducted a multi‐location common garden experiment using *B. ermanii* to (1) clarify the contribution of both environmental and genetic factors to the variation in stomatal density and size of *B. ermanii*, (2) demonstrate the differences in the plasticity of stomatal density and size among *B. ermanii* populations, and (3) understand how stomatal density and size of *B. ermanii* would respond to increased temperature and changing precipitation patterns. Genetic factors played a more significant role in stomatal size than environmental factors, suggesting that *B. ermanii* struggles to adjust its stomatal size in response to a changing environment. Our results also revealed a positive correlation between stomatal size plasticity and original habitat suitability, indicating that in *B. ermanii* populations in harsh environments exhibit lower adaptability to environmental shifts. Although stomatal density and size of *B. ermanii* showed the significant responses to increased temperature and shifting precipitation patterns, the response ranges of stomatal density and size to the environmental factors varied among populations. Our findings highlighted the interplay between genetic and environmental factors in determining the intraspecific variation in stomatal density and size in *B. ermanii*. This indicated that certain populations of *B. ermanii* exhibit limited stomatal plasticity and adaptability, which could directly affect photosynthesis and transpiration, suggesting potential population‐specific fitness implications for *B. ermanii* under future climate change.

## Graphical abstract

A multi‐location common garden experiment revealed that stomata density was equally determined by both genetic and environmental factors, while genetic factors played a more significant role in stomatal size than environmental factors. Additionally, it became clear that the plasticity of stomatal size was lower in populations from relatively harsh environments, and the responses of stomatal density and size to mean annual temperature, annual precipitation, and aridity index were different among populations. These results indicate the possibility of population‐specific fitness implications for *B. ermanii* under future climate change scenarios.

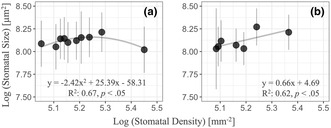

## 1 INTRODUCTION

As plants cannot move, their distribution and performance are determined by both the surrounding environmental factors and their adaptive potential, that is, genetic factors (Anderson & Gezon, 2015; Bertolino et al., 2019; Reich, 2014). Thus, clarifying the effects of these two factors is key to understanding the ability of plants to respond to the environment and predicting their distribution under ongoing global warming. The best materials for such studies are species that are currently distributed across a wide range of environments, with studies demonstrating their ability to change ecological traits to adapt to the environment in which they are placed. *Betula ermanii* is a species that exactly meets these conditions. It is a wind‐pollinated deciduous tree species distributed in cool, snowy environments across eastern Russia, northern China, the Korean Peninsula, and Japan (Ashburner & McAllister, 2013). In Japan, it has a large distribution near timberlines in mountainous areas (Takahashi et al., 2005) and is thought to be particularly affected by recent global warming in some regions and populations. Indeed, recent comprehensive studies of leaf traits and phenology published by Paing et al. (2021) and Aihara et al. (2024) reported that *B. ermanii* is under strong selection pressure from changing rainfall patterns and increasing air temperatures associated with the ongoing climate change and that this pressure varies among populations. However, it is still unknown how other critical functional traits that affect plant adaptation to the climate change and whether the responses of these functional traits differ among population of the same species.

Stomata are fundamental gatekeepers on the surface of leaves, flanked by guard cells that regulate the gas exchange between plants and the atmosphere, especially water vapor and CO_2_ (Brodribb & McAdam, 2017; Zhang et al., 2012). Stomatal anatomical traits, such as stomatal density and size, which strongly control the gas and water exchange rate, are considered one of the primary factors in the plant's photosynthetic and transpiration processes (Fraser et al., 2008; Wu et al., 2018). In general, small stomata can be closed more quickly than larger stomata (Drake et al., 2013; Poulos et al., 2007); therefore, they are more suitable for survival and growth under water‐deficit conditions. According to the trade‐off relationship between stomatal size and density (Du et al., 2021; Wang et al., 2007), smaller stomatal is along with higher stomatal density that have been reported to show higher photosynthetic rate (Xu & Zhou, 2008). However, some studies have demonstrated the opposite results that lower stomatal density improved water use efficiency (Caine et al., 2019; Petrík et al., 2023) and lead to higher photosynthesis rate (Guo et al., 2019). In addition to temperature and precipitation pattern‐induced heterogeneous environment, altitude with different CO_2_ and O_2_ partial pressures can also show the significant impact on stomatal density and size (Wang et al., 2014). Some studies showed the increase in stomatal density with rising altitude that leads to thinner CO_2_ concentration (Liu et al., 2020), while other studies showed that this response could be species‐specific (Kessler et al., 2007). Considering the vital role of stomata in plant physiological processes closely related to plant survival and growth (Bucher et al., 2017; Vatén & Bergmann, 2013), a better understanding of how the anatomical traits of *B. ermanii* stomata respond to changing environments will provide deeper insights into the adaptation of *B. ermanii* to a changing climate.

In addition to the environmental factors, genetic factors are also the important aspect to determine stomatal anatomical traits (Casson & Hetherington, 2010). Some species are more sensitive to environmental changes, whereas others have higher heritability and change less with varying environments (Zhang et al., 2012). However, the relative importance of genes versus the environment in determining stomatal anatomical traits in *B. ermanii* has not yet been estimated across populations and a wide range of environments. In addition, even within the same species, the likelihood of phenotypic plasticity among different populations can vary depending on the traits, present and previous environment (Thakur et al., 2023), leading to different adaptation levels of stomatal anatomical traits under future climate change. According to previous studies, populations originally inhabiting relatively intense environments have relatively low trait plasticity, as they cannot afford the cost of maintaining the genetic and cellular machinery necessary to be plastic (Falconer, 1990; Scheiner, 1993); therefore, they could be more sensitive to changing environments.

The present study had three objectives: (1) to clarify whether genetic or environmental factors are more important in determining the variation in stomatal density and size of *B. ermanii*; (2) to demonstrate the difference in the plasticity of stomatal density and size among *B. ermanii* populations; and (3) to understand how stomatal density and size of *B. ermanii* would respond to both increased temperature and changing precipitation patterns. To investigate this, we conducted a multi‐location common garden experiment using populations throughout the distribution area of *B. ermanii* in Japan. Based on previous studies showing that leaf traits in *B. ermanii* were rather strongly influenced by environmental factors, we hypothesized that stomatal density and size would also be controlled by environmental factors. We expected that stomatal density and size of *B. ermanii* from different populations would show different plasticities related to the environment of the original habitat. Specifically, populations inhabiting harsher environments are expected to demonstrate lower plasticity in both stomatal density and size. Regarding the response to different environments, we hypothesized that stomatal density would be higher and stomatal size would be smaller in drier environments, whereas the opposite would be true in wetter environments.

## 2 METHODS

### 2.1 Locality description and climatic conditions

**FIGURE 1:**
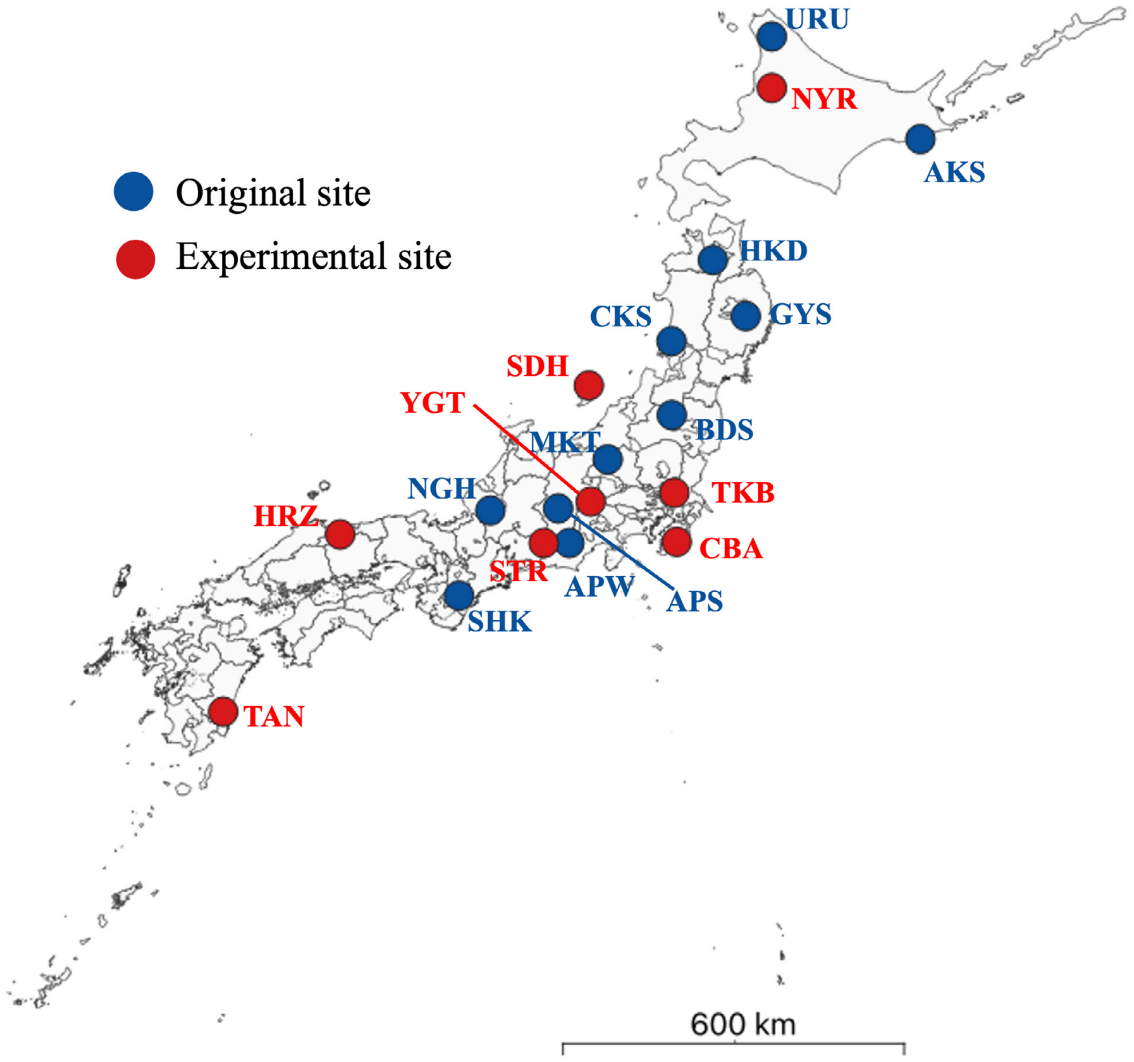
Location of the original sites (blue) and experimental sites (red).

From 2016 to 2017, seeds of *B. ermanii* were collected from 11 natural source populations throughout its distribution zones in Japan: Uryu (URU), Akkeshi (AKS), Hakkoda (HKD), Goyo‐San (GYS), Choukai‐San (CKS), Bandai‐San (BDS), Mikuni‐Touge (MKT), Alps‐West (APW), Nougouhaku‐San (NGH), Alps‐South (APS), and Shakaga‐Take (SHK) (Figure 1; Table 1). For each population, seeds were collected from 7 to 15 mother trees (Paing et al., 2021). In April 2018, after collecting a set of seeds (2000 seeds from each tree), the seeds were mixed and sown in a nursery located at the University of Tokyo Hokkaido Forest in Furano, central Hokkaido, Japan (43°13′10″ N, 142°22′55″ E). In June of the same year, the newly germinated saplings were transferred to 150 cm^3^ JFA containers (Japan Forest Agency, 2009) and cultivated in a greenhouse for two successive growing seasons in 2018 and 2019. Finally, in autumn 2019 or spring 2020, containerized saplings were planted at eight experimental sites: Nayoro (NYR), Sado High Altitude (SDH), Tsukuba (TKB), Yatsugatake (YGT), Hiruzen (HRZ), Chiba (CBA), Shitara (STR), and Tano (TAN) (Figure 1; Table 1). Except for GYS (10 saplings), AKS (nine saplings), and CKS (four saplings), 20 saplings were planted from each of the remaining eight seed source populations, resulting in a total of 183 saplings per planting site. More comprehensive information about the common garden experiment, such as the planting arrangement of the saplings and soil variables at each planting site, can be found in Paing et al. (2021) and Aihara et al. (2023).

**TABLE 1:** Basic information about the experimental sites and original sites.

	ID	Latitude	Longitude	Altitude (m)	MAT (°C)	Precipitation (mm)	AI	SD (μm^−2^)	SS (μm^2^)
Experimental site	TAN	31.85	131.31	130	17.4	2820	104.9	146.12 (25.81)	529.94 (158.31)
	STR	35.14	137.55	684	11.5	1964	134.5	218.46 (46.25)	442.44 (101.14)
	CBA	35.16	140.15	294	14	2200	86.1	177.20 (31.51)	473.26 (113.78)
	HRZ	35.3	133.59	590	11.2	2010	108.5	168.67 (37.84)	513.01 (95.35)
	YGT	35.94	138.47	1350	7.1	1454	78.3	169.49 (50.44)	528.56 (136.51)
	TKB	36.12	140.1	30	13.8	1196	52.2	166.71 (37.58)	496.52 (120.69)
	SDH	38.21	138.43	800	11.4	1800	113.2	194.72 (49.45)	527.59 (105.27)
	NYR	44	142	98	5.5	969	61.2	182.67 (32.47)	437.35 (80.50)
Original site	SHK	34.11	135.9	1780	13.2	2714	181.1	238.91 (52.63) a	334.23 (70.72) a
	APS	35.14	138.05	1450	7.6	1956	169.1	202.22 (44.36) b	473.36 (80.28) b
	NGH	35.77	136.51	1503	12.7	3229	173.2	185.97 (34.61) bc	487.33 (98.80) bc
	APW	35.81	137.84	2453	1.1	1542.9	233.4	174.34 (35.62) cd	532.46 (114.66) c
	MKT	36.77	138.8	1304	5.62	1743	104.2	177.02 (35.39) c	486.68 (84.38) bc
	BDS	37.63	140.05	1076	5.7	1887	101.1	171.75 (35.41) cd	527.11 (122.27) c
	CKS	39.07	140.05	1205	3.27	2619	182.5	182.54 (38.31) bcd	488.96 (72.61) bc
	GYS	39.56	141.49	1795	7.4	1062.4	103.5	191.93 (38.50) bc	487.74 (105.16) bc
	HKD	40.65	140.85	898	4.7	1624	130.8	159.76 (32.90) de	540.91 (137.04) c
	AKS	43	144.9	43	5.5	1114	70.1	168.35 (31.82) cde	502.38 (119.56) bc
	URU	45	142	290	3	1400	84.3	151.10 (38.86) e	532.78 (128.38) c

*Note*: MAT, AI, SD, and SS represent the MAT, aridity index, stomatal density, and stomatal size, respectively. Small letters in the table indicated the results of multi‐comparison of stomatal density and size among experimental sites and original sites.

The mean meteorological data for the 10‐year period from 2010 to 2020 at each site location were obtained from the Agro‐Meteorological Grid Square Data (https://amu.rd.naro.go.jp). The data had a spatial resolution of approximately 1 km × 1 km and were used to complement the meteorological dataset of both the experimental and original sites. The following meteorological indexes were calculated as climatic variables: mean annual temperature (MAT; °C), mean temperature in summer (May–September) (TS), annual precipitation (PRT; mm), precipitation in summer (May–September) (PRS), and aridity index (AI); AI was calculated from MAT and PRT. A low AI indicates relatively dry climatic conditions, whereas a high AI indicates relatively wet climatic conditions. Habitat suitability for *B. ermanii* in Japan was predicted using the maximum entropy principal algorithm in MaxEnt (Phillips & Dudík, 2008). Detailed information on this estimation can be found in Aihara et al. (2024).

### 2.2 Stomatal anatomical traits

Samples were taken from full sun‐exposed leaves of every live sapling at each experimental site in the period from June to August 2021. As *B. ermanii* has both early leaves which unfold around early May and late leaves which begin unfolding in late May or early June, we only used the early leaves because of their more stable phenotypes (Kozlowski & Clausen, 1966; Tabata et al., 2010) and to avoid undesired noise in stomatal anatomical traits data. The nail‐polish imprint method (Pyakurel & Wang, 2014; Zhao et al., 2016) was used to measure stomatal density and size on the abaxial side of leaves as woody plants generally have hypostomatous leaves (stomata on the abaxial side) (Peat & Fitter, 1994). Leaves were coated with a clear and transparent nail polish, carefully avoiding the major veins, and the polish was allowed to dry naturally. The polish was then removed from the leaves and mounted on glass slides. The number of stomata, stomatal length, and stomatal width were measured at 400× magnification using an attachable high‐solution camera (AdvanCam‐E3H, Tokyo, Japan) mounted on a microscope (BX53; Olympus, Tokyo, Japan). Each picture was projected on the computer using the AdvanView imaging software (version 3.7). Therefore, we could easily count the number of stomata and measure stomatal length and width. The number of stomata was counted within the 0.04 mm^2^ measured imaged area, and the stomatal length and width were measured three times in each of the imaged areas (Figure S1). Three image areas were randomly selected from each sample. The stomatal density (mm^−2^) and size (μm^2^, Xie et al., 2022) were calculated as follows:

(1) $$\text{stomatal density}=\frac{\text{number of stomata}}{0.04}$$

(2) $$\text{stomatal size}=\text{stomatal length}\times \text{stomatal width}\times \frac{\pi }{4}$$

As *B. ermanii* seeds were planted at all eight experimental sites collected from the 11 original sites with 20 individuals each (except for the GYS, AKS, and CKS sites with 10, 9, and 4 individuals, respectively), a total of 1464 samples were expected to be collected. As some seedlings died during the first year, 908 samples were collected in the end.

### 2.3 Calculation of the plasticity index of stomatal density and size

The plasticity index (PI) based on maximum and minimum means was used to quantify the plasticity for both stomatal density and site for each original site, respectively (Petrík et al., 2020; Valladares et al., 2006).

(3) $$\mathrm{PI}=\frac{{\bar{X}}_{\max}-{\bar{X}}_{\min}}{{\bar{X}}_{\max}}$$

where $\bar{X}$_max_ is the greatest mean stomatal density or size among the experimental sites from the same original site, and $\bar{X}$_min_ is the lowest mean stomatal density or size among the experimental sites from the same original site.

### 2.4 Statistical analysis

The stomatal density and size proportion data were log‐transformed prior to each analysis to follow a normal distribution (McDonald, 2014). Differences in stomatal density and size among the experimental and original sites were tested with one‐way ANOVA and multiple comparisons using the “multcompView” package (Graves et al., 2023). Genetic and environmental effects on stomatal anatomical traits were tested using ANOVA‐type estimation of variance components. The experimental site, original site, and their interactions were used as the fixed factors. The F value using Fisher's test and the ratio of the variance component were calculated using the “VCA” package in R (Andre & Florian, 2022). Statistical significance of the differences between the plasticity of stomatal density and size was assessed using analysis of variance and Tukey's post‐hoc test. To evaluate the relationship between the habitat flexibility and plasticity of stomatal density and size of original sites, both linear and quadratic regression models were run using the “lme4” package in R (Bates et al., 2015). Multi‐regression was conducted with climatic factors (MAT, TS, PRT, PRS, AI, altitude) of both experimental and original sites as explanatory factors to explain stomatal density and size. Linear regression was conducted to see the relationship between eco‐distance (differences between experimental site and original site) of climatic factors and stomatal density and size. A *p*‐value <.05 was considered statistically significant, and all statistical analyses were performed using R 4.0.4 (R Development Core Team, 2022).

## 3 RESULTS

### 3.1 Characteristics of stomatal density and size among experimental site and original site

**FIGURE 2:**
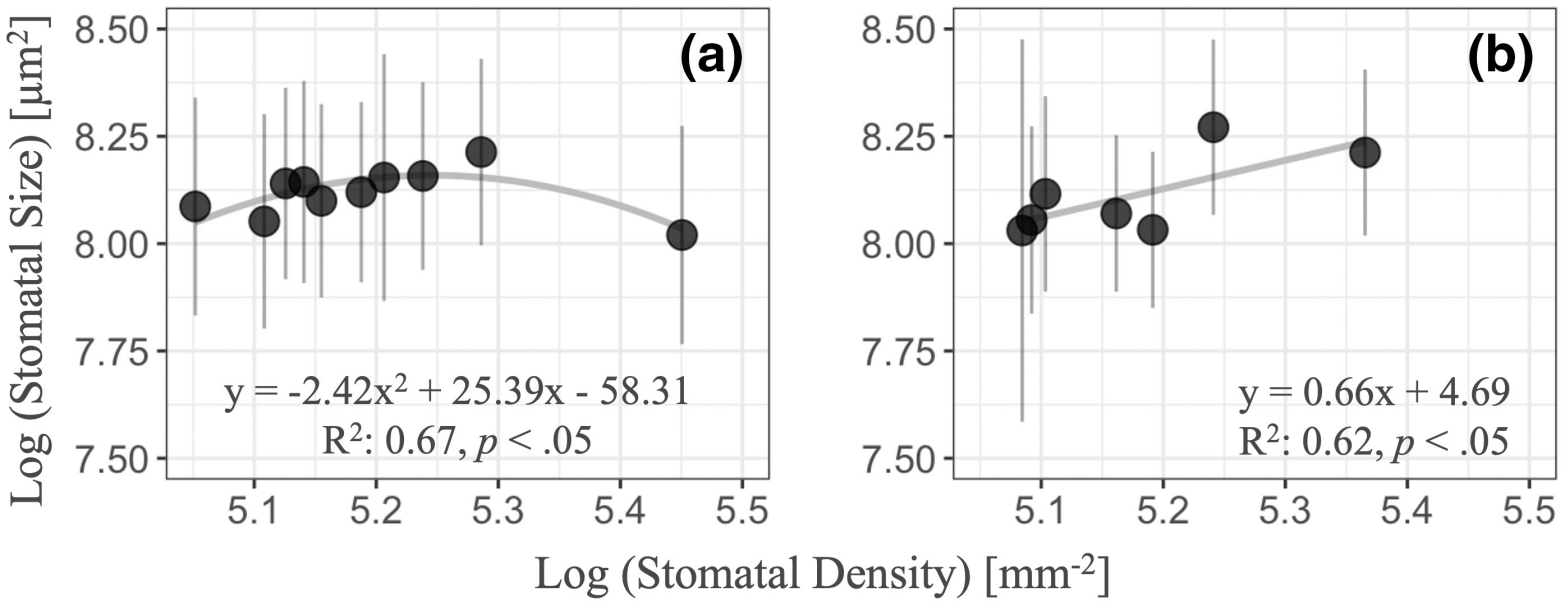
Relationships between stomatal density and stomatal size as original site (a), and as experimental site (b).

Significant differences in stomatal density and size were detected among experimental site (*p* < .001) and original site (*p* < .001). Among experimental sites, stomatal density was the highest at the STR and stomatal size was the greatest at the TAN. Among original sites, stomatal density was highest at the SHK, and stomatal size was the greatest at the HKD (Table 1). Significant quadric correlation was detected between stomatal density and size based on original site (Figure 2a). And significant positive linear correlation was detected between stomatal density and size based on experimental site (Figure 2b), which indicated there was no trade‐off relationship between stomatal density and stomatal size of *B. ermanii* among experimental sites.

### 3.2 Contribution of genetic and environmental factors and the plasticity of stomata anatomical traits from different populations

**FIGURE 3:**
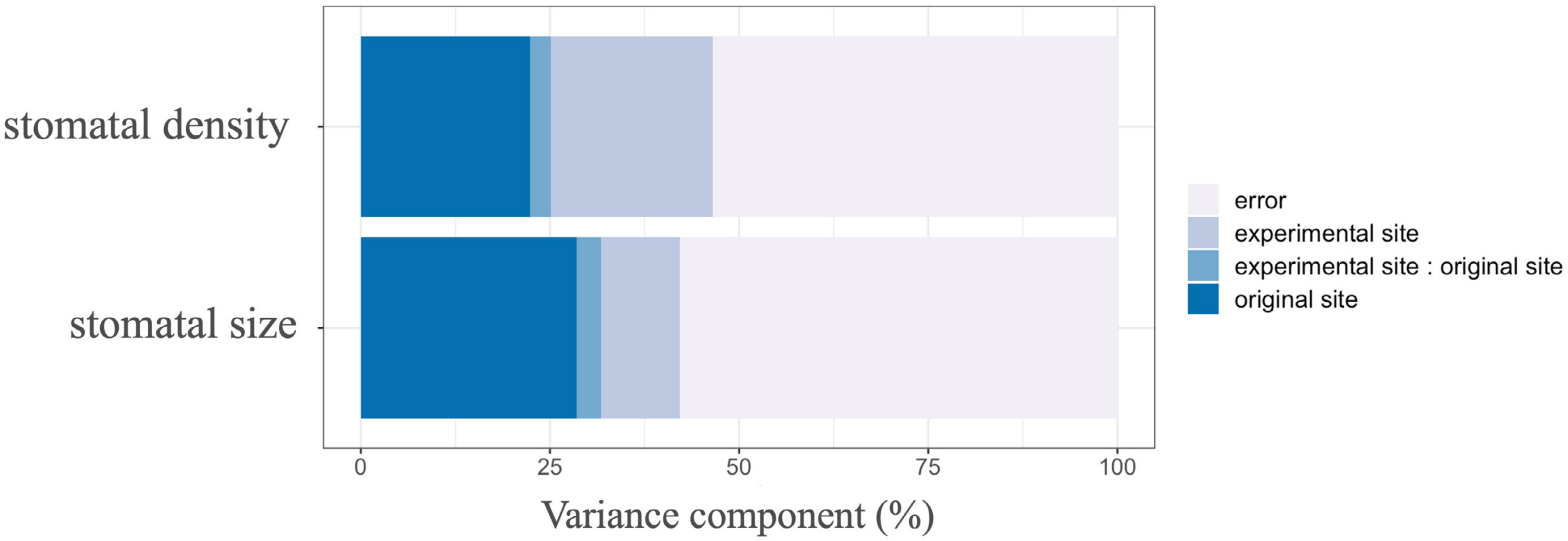
Contribution of each experimental site, original site, and the interaction between experimental site and original site to the stomatal density and size according to ANOVA. The error indicates the residuals of ANOVA. Percentages of total variance explained are the variance components reported in Table S1.

The experimental site (environment), original site (genetics), and their interactions significantly affected both stomatal density and size (Figure 3). Both the environment and genetics showed similar relative importance for stomatal density. However, for stomatal size, the relative importance of genetics was much greater than that of environmental factors. Also, climatic factors of both experimental sites and original sites were selected to explain the stomatal density and size through the results of multi‐regression (Table 2). Significant negative effects of altitude and mean temperature in summer (May–September) of experimental site (Site_Altitude, Site_TS), and positive effects of aridity index of experimental site (Site_AI) and mean temperature in summer (May–September) and annual precipitation of original site (Pop_TS, Pop_PRT) were detected on stomatal density (Table 2a). Opposite effects were detected on stomatal size, namely significant positive effects of Site_Altitude and Site_TS, and negative effects of Pop_TS and Pop_PRT (Table 2b).

**TABLE 2:** The result of the multi‐regression to explain stomatal density (a) and size (b).

	Estimate	Std. error	*t*‐value	*p*‐value
*(a)*				
Intercept	4.687	0.093	50,378	<.001
Site_Altitude	−0.000	0.000	−2.866	<.01
Site_TS	−0.017	0.003	−5.506	<.001
Site_AI	0.003	0.000	7393	<.001
Pop_TS	0.017	0.005	3.259	<.01
Pop_PRT	0.000	0.000	12.062	<.001
Multiple *R* ^2^: .22	Adjusted *R* ^2^: .21		*p*‐value: <.001	
*(b)*				
Intercept	6.374	0.092	68.956	<.001
Site_Altitude	0.000	0.000	6.615	<.001
Site_TS	0.014	0.003	4.328	<.001
Site_AI	−0.000	0.000	−1.576	.115
Pop_TS	−0.016	0.005	−3.050	<.01
Pop_PRT	−0.000	0.000	−8.938	<.001
Multiple *R* ^2^: .13	Adjusted *R* ^2^: .13		*p*‐value: <.001	

*Note*: Site_Altitude, altitude of experimental site; Site_TS, mean temperature in summer (May–September) of experimental site; Site_AI, aridity index of experimental site; Pop_TS, mean temperature in summer (May–September) of original site; Pop_PRT, annual precipitation of original site.

**FIGURE 4:**
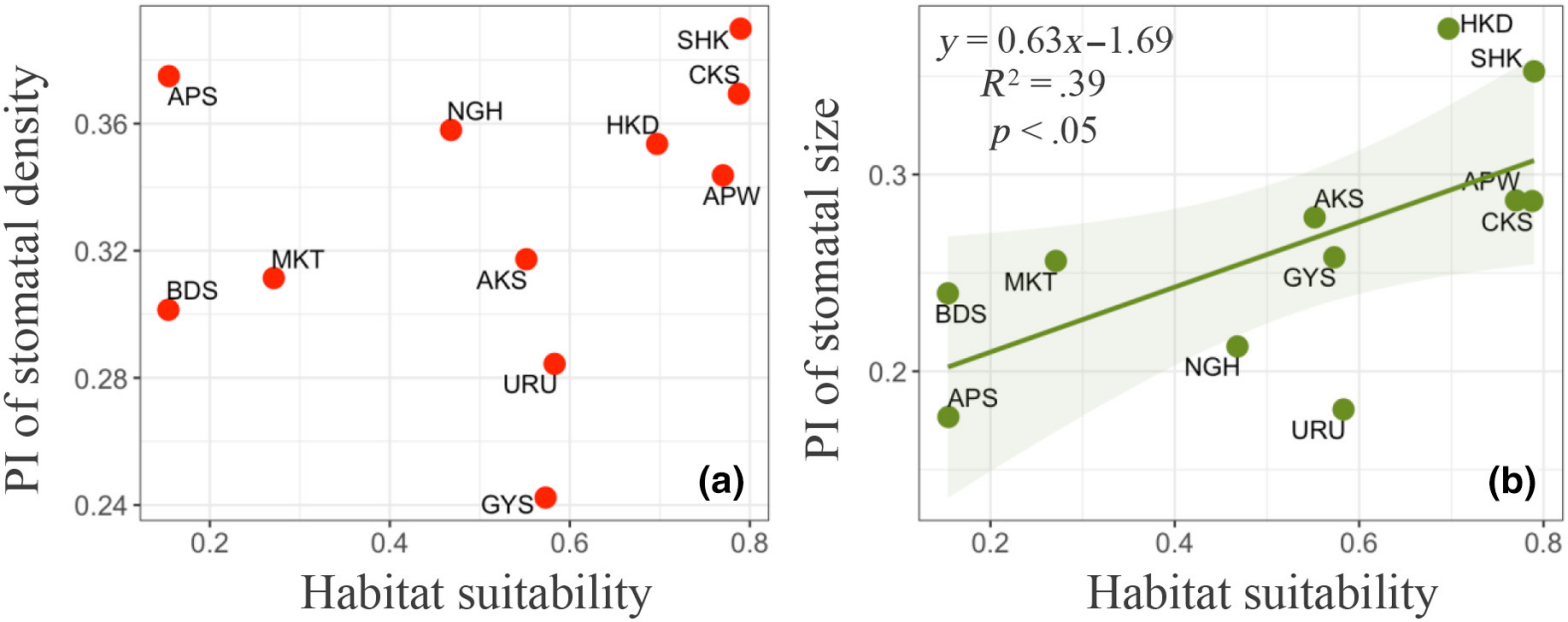
Relationships between habitat suitability and plasticity indices (PI) of stomatal density (a) and size (b).

For stomatal density, SHK (0.39) had the greatest phenotypic plasticity, while GYS (0.24) had the lowest phenotypic plasticity (Figure 4a). And for stomatal size, HKD had the greatest phenotypic plasticity, while APS had the lowest phenotypic plasticity (Figure 4b). No significant correlation was found between habitat suitability and the PI (plasticity index) of stomatal density (Figure 4a), whereas a significant positive correlation was found between habitat suitability and the PI of stomatal size (Figure 4b, *p* < .05). In addition, stomatal density showed a significantly higher PI than that of stomatal size (Figure S2, *p* < .01).

### 3.3 Responses of stomatal density and size to changing environments

**FIGURE 5:**
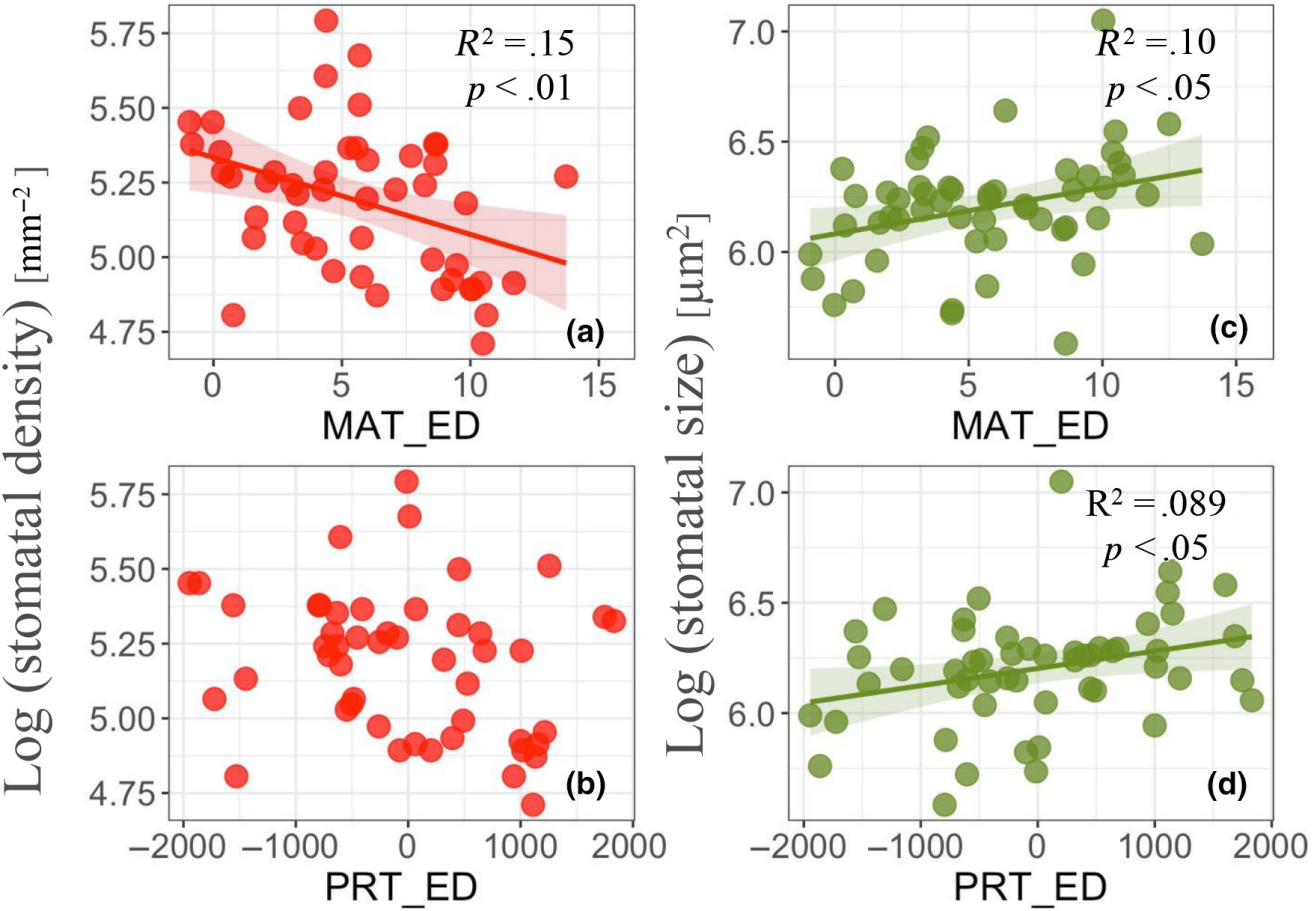
Linear regression results showing the relationship between eco‐distance (MAT_ED, PRT_ED) and stomatal density (a, b) and stomatal size (c, d). Positive values on the *x*‐axis represent transfer to a hotter environment, and negative values represent transfer to a colder environment (a, c). Also, positive values on the *x*‐axis represent transfer to a more humid environment, and negative values represent transfer to a drier environment (b, d).

The degree of temperature and precipitation differences between experimental sites and original sites (eco‐distance) had the significant effects on stomatal density and size (Figure 5; Figure S4). Stomatal density decreased with increased temperature (MAT [*y* = −0.03*x* + 5.33, *R* ^2^ = .15, *p* < .01], TS [*y* = −0.03*x* + 5.22, *R* ^2^ = .11, *p* < .05]) (Figure 5a,c) and stomatal size increased with temperature (MAT [*y* = 0.02*x* + 6.08, *R* ^2^ = .10, *p* < .05]) (Figure S4a). Decreased precipitation (PRS) increased stomatal density, while increased precipitation decreased stomatal density (*y* = −0.0001*x* + 5.20, *R* ^2^ = .10, *p* < .05) (Figure S5d). Also, decreased precipitation (PRT, PRS) decreased stomatal size, while increased precipitation increased stomatal size (PRT [*y* = 0.0001*x* + 6.20, *R* ^2^ = .089, *p* < .05], PRS [*y* = 0.0001*x* + 6.18, *R* ^2^ = .14, *p* < .01]) (Figure 5a,c,d; Figure S4d).

The responses of stomatal density to MAT among the different populations were different, such that AKS, GYS, and URU sites had no significant relationships, and CKS had a significant linear correlation with MAT, and other sites had the significant quadratic correlations (Figure S5). Regarding the AP, only AKS, APS, BDS, NGH, and SHK had the significant correlations, while other six original sites did not show the correlations (Figure S6). For stomatal size, only four sites, CKS, HKD, MKT, and NGH, showed significant correlations with MAT that CKS, MKT, and NGH had significant quadratic correlations and HKD had significant linear correlation (Figure S5). Lastly, significant quadratic correlations between AP and stomatal size were detected at the AKS, APS, BDS, HKD, and MTK sites, and significant linear correlations between AP and stomatal size were detected at the NGH site (Figure S8).

## 4 DISCUSSION

### 4.1 Genetic contribution to stomatal anatomical traits of *B. ermanii*

*B. ermanii* from different populations showed variations in stomatal density and size (Table 1). There was a trend indicating higher stomatal density in populations from lower latitudes, while populations from higher latitudes exhibited larger stomatal size which consisted of general trend (Hill et al., 2014; Wang et al., 2015). However, we need to note that the southern boundary populations of *B. ermanii* (referred to as SHK in this paper) were found to be diploid, which differs from other tetraploid populations (T. Aihara, K. Araki and Y. Tsumura, unpublished data). Therefore, the highest stomatal density combined with smallest stomatal size of SHK *B. ermanii* matches the general trend observed that tetraploid usually has larger and fewer stomata than diploids (Li et al., 1996; Pacey et al., 2022).

In the present study, the relative importance of the environment (experimental site) and genetics (original site) in determining stomatal density and size was quantitatively estimated. Comprehensive stomatal observations suggested that genetic factors are the primary regulators of stomatal density and stomatal size in *B. ermanii* and that they, especially stomatal size, are much more important than environmental factors (Figure 2). These results consisted of previous studies, showing that environmental factors had a weaker effect on stomatal size than genetic factors did (Čortan et al., 2017; Yan et al., 2017; Zhang et al., 2012). One reason why genetic factors had a larger contribution to stomatal size than environmental factors may be related to the fact that geologic time‐scale CO_2_ variability strongly controls stomatal size (Franks & Beerling, 2009). And this result implied that it is easier for plant to alter their stomatal density to adapt to the changing temperature and precipitation patterns compared to alter stomatal size. The fact that the PI of stomatal density was greater than the PI of stomatal size also suggested that stomatal density can change more variable than stomatal size under changing environmental conditions (Figure S2). The PI of stomatal size was positively correlated with habitat suitability of the original population, whereas no correlation was detected between habitat suitability of the original population and the PI of stomatal density, which was partly consistent with our hypothesis (Figure 3). The lower PI of stomatal size found in populations with stronger environmental selection was consistent with the suggestion of previous studies (Falconer, 1990; Scheiner, 1993). These results suggested that *B. ermanii* populations currently growing under strong environmental selection may experience difficulties in altering the anatomical traits of the stomata, which directly affect the photosynthetic and transpiration processes of the plant under changing environmental conditions. In addition, compared to certain leaf traits of *B. ermanii* such as leaf area and specific leaf area (Aihara et al., 2024), stomatal density and size were more easily determined by genetics, indicating that it is more difficult for *B. ermanii* plants to change their stomatal anatomical traits than to change their leaf morphological traits. The relatively higher heritability of stomatal anatomical traits compared to leaf traits would be because stomata only consist of few cells and therefore, more easily influenced by genetics.

### 4.2 Response of stomatal density and size of *B. ermanii* to changing environments

Through the multi‐regression, it became clear that temperature could explain both stomatal density and size much more than other selected factors such as precipitation, aridity index, and altitude (Table 2). The correlations between eco‐distance of temperature (MAT, TS) and the experimental sites indicated that stomatal density of *B. ermanii* would decrease under rising temperature condition (Figure 5; Figure S4) which consisted of previous study focused on European beech (Petrík et al., 2020). The decreased stomatal density that can prevent water loss (Hepworth et al., 2015) from *B. ermanii* seedling in warmer sites, however, would decrease their photosynthesis rate and overall growth correspondingly (Xu & Zhou, 2008). The decline of both stomatal density and size to both higher and lower temperatures was detected in some of the population (Figures S5 and S7). Both declines to higher temperature can be seen as acclimation response to reduce transpiration under higher vapor pressure deficit. On the other hand, the decline of both stomatal density and size to lower temperature may be related to photosynthetic limitation under the lower temperature. As plants cannot utilize much CO_2_ under low‐temperature conditions, they decrease both stomatal density and size to optimize water use efficiency instead. The negative correlation between eco‐distance of PRS and stomatal density of *B. ermanii*, and positive correlation between eco‐distance of precipitation (PRT, PRS) and stomatal size of *B. ermanii* were also detected (Figure 5; Figure S4). The small stomata have an advantage over large stomata in dry habitats because of their quick open and close responses (Franks et al., 2009; Hetherington & Woodward, 2003; Yin et al., 2020). In addition, the combination of higher stomatal density, which reduces the CO_2_ gas molecule diffusion resistance (Bosabalidis & Kofidis, 2002; Liu et al., 2019, 2020), could increase the maximum stomatal conductance and lead to higher photosynthesis rates (Franks & Beerling, 2009). Thus, *B. ermanii* has a higher density of smaller sized stomata in relatively dry sites, which will contribute to water retention within *B. ermanii* leaves, as well as maximizing the rate of photosynthesis. The increase in stomatal density and decrease in stomatal size were also detected once after precipitation reach to certain degree (Figures S6 and S8). However, the small stomata that appear at high precipitation sites may not be selected specifically to cope with the climate; they could be selected as a defense against pathogens (McKown et al., 2014; Xie et al., 2022). Stomata, as gatekeepers for the exchange of gas and water, could also be an entryway for foliar pathogens into plant tissues (Melotto et al., 2008). Because of the greater threat of foliar pathogens in relatively wet regions, smaller stomata might be selected to reduce the entry of foliar pathogens in these experimental sites (Muir, 2020). However, stomatal density was higher in the relatively wet experimental sites, which could increase the possibility of pathogen colonization. Considering the shortage of empirical studies to reveal how pathogen defense influences stomatal anatomical traits and how it interacts with abiotic factors and affects the stomatal anatomical traits, more studies are still necessary.

Through the analysis to see how the responses of stomatal density and size to shifting temperature and precipitation differ among original population, it became clear that not all *B. ermanii* populations had similar stomatal density and size responses to shifting environment (Figures S5–S8). Specifically, some *B. ermanii* populations, such as AKS, CKS, GYS, and URU, showed little change in stomatal density or size in response to changes in MAT and PRT. The different responses among populations imply the possibility that certain populations of *B. ermanii* may lack the ability or may not need to change their stomatal density and size to adapt to the environment. In case that populations of *B. ermanii* are unable to alter their stomatal anatomical traits due to low plasticity caused by the harsh conditions of their original habitat, the changing environment could significantly negatively impact their fitness. On the other hand, for populations of *B. ermanii* that did not alter their stomatal anatomical traits considerably, it is possible for them to adjust leaf mass per area (Li et al., 2021; Poorter et al., 2009), leaf vein density (Zhu et al., 2012), or other functional traits to adapt to the environment (Xie et al., 2022). Therefore, a comprehensive investigation of functional traits, conducted simultaneously to reveal the interconnections between these traits for environmental adaptation combined with the survival, growth, and reproduction performance in a long term, is necessary.

## 5 CONCLUSION

Our results clearly showed that both stomatal density and size were significantly affected by both genetic and environmental factors, with genetic factors explaining stomatal size much more than environmental factors did. It also became clear that both stomatal density and size of *B. ermanii* could be affected by both experimental sites and original sites' environmental factors, and the plasticity of stomatal size was determined by the suitability of the original habitat. Through the linear regression between eco‐distance of climatic factors and stomatal density and size, we observed the consistent responses that stomatal density showed the decrease while stomatal size showed increase to increased temperature and precipitation. However, when we look into the relationships between stomatal anatomical traits and environmental factors in the original population, different responses were detected among the populations. As the stomata regulate gas and water exchange, and anatomical traits directly influence plant photosynthesis and transpiration rates, our study provides important information for explaining the different adaptations (survival and growth rate) among *B. ermanii* populations under changing environments. However, long‐term observations and studies that more comprehensively measure functional traits related to environmental adaptation are still necessary.

## AUTHOR CONTRIBUTIONS

**Yihan Cai:** Data curation (lead); formal analysis (lead); funding acquisition (equal); investigation (equal); methodology (equal); software (lead); validation (lead); visualization (lead); writing – original draft (lead). **Takaki Aihara:** Data curation (equal); investigation (equal); methodology (equal); software (supporting); writing – review and editing (supporting). **Kyoko Araki:** Investigation (equal); writing – review and editing (supporting). **Ragini Sarmah:** Investigation (equal); writing – review and editing (supporting). **Yoshihiko Tsumura:** Conceptualization (equal); funding acquisition (lead); investigation (equal); project administration (lead); resources (equal); writing – review and editing (supporting). **Mitsuru Hirota:** Conceptualization (equal); methodology (equal); resources (equal); supervision (equal); writing – review and editing (lead).

## FUNDING INFORMATION

This study was supported by the JSPS KAKENHI program (grant no. 21H04732) and JST SPRING (Grant Number JPMJSP2119, JPMJSP2124).

## CONFLICT OF INTEREST STATEMENT

The authors declare no conflict of interest.

## Supporting information


Data S1

## Data Availability

The original data are forthcoming on Dryad https://doi.org/10.5061/dryad.ttdz08m5d.
