# Supplementary material for: Response of stomatal density and size in Betula ermanii to contrasting climate conditions: The contributions of genetic and environmental factors
Source: Ecol Evol. 2024 Jun 18;14(6):e11349. doi: 10.1002/ece3.11349 (PMC11184283; doi:10.1002/ece3.11349)
Supplement: Supplementary file 1 — Data S1 [file ECE3-14-e11349-s001.docx]

Fig. S1. Images of stomata on the abaxial leaf surface of *Betula ermanii* captured with a high-resolution camera (AdvanCam-E3H) mounted on a microscope (Olympus BX53) at 400× magnification.

Fig. S2 Plasticity index (PI) of stomatal density and size.


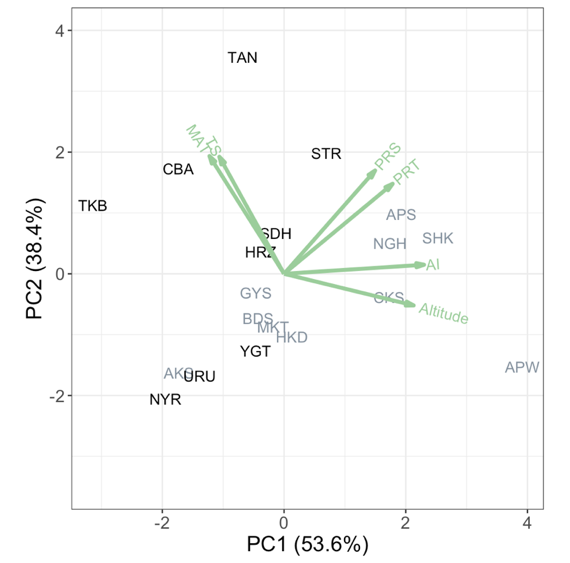


Fig. S3 Results of principal component analysis of original site (black letter) and experimental sites (grey letter) with climatic variables. Green characters indicate climatic variables used for the analysis. Abbreviations: MAT, mean annual temperature; TS, mean temperature in summer (May–Sept.); PRT, annual precipitation; PRS, summer precipitation (May–Sept.); Altitude.

Fig. S4 Linear regression results showing the relationship between eco-distance (TS_ED, PRS_ED) and stomatal density (a, b) and stomatal size (c, d). Positive values on the x axis represent transfer to a hotter environment, and negative values represent transfer to a colder environment (a, c). Also, positive values on the x axis represent transfer to a more humid environment, and negative values represent transfer to a drier environment (b, d).


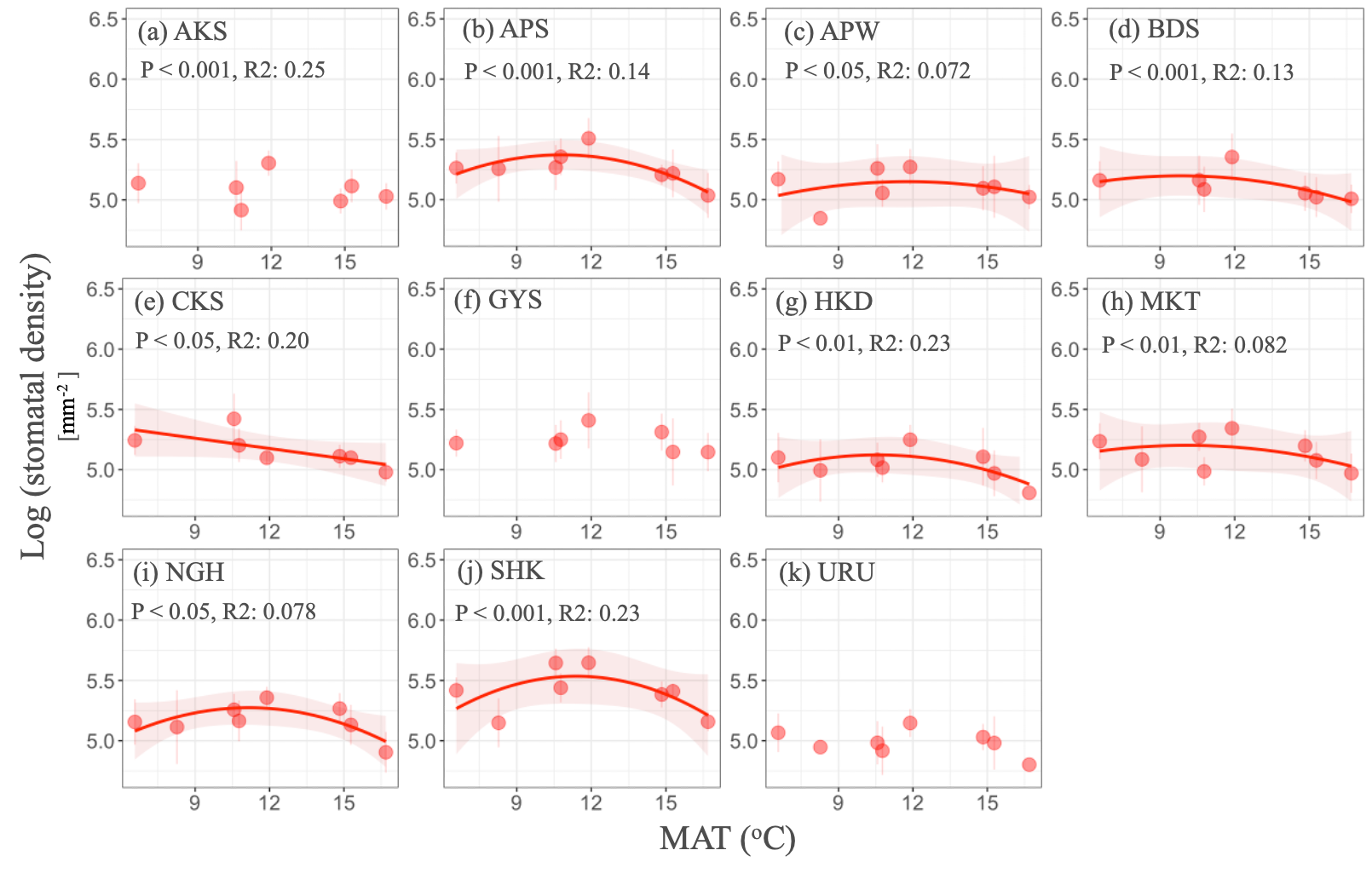


Fig. S5 Relationship between mean annual temperature (MAT) of experimental sites and stomatal density of different original sites.


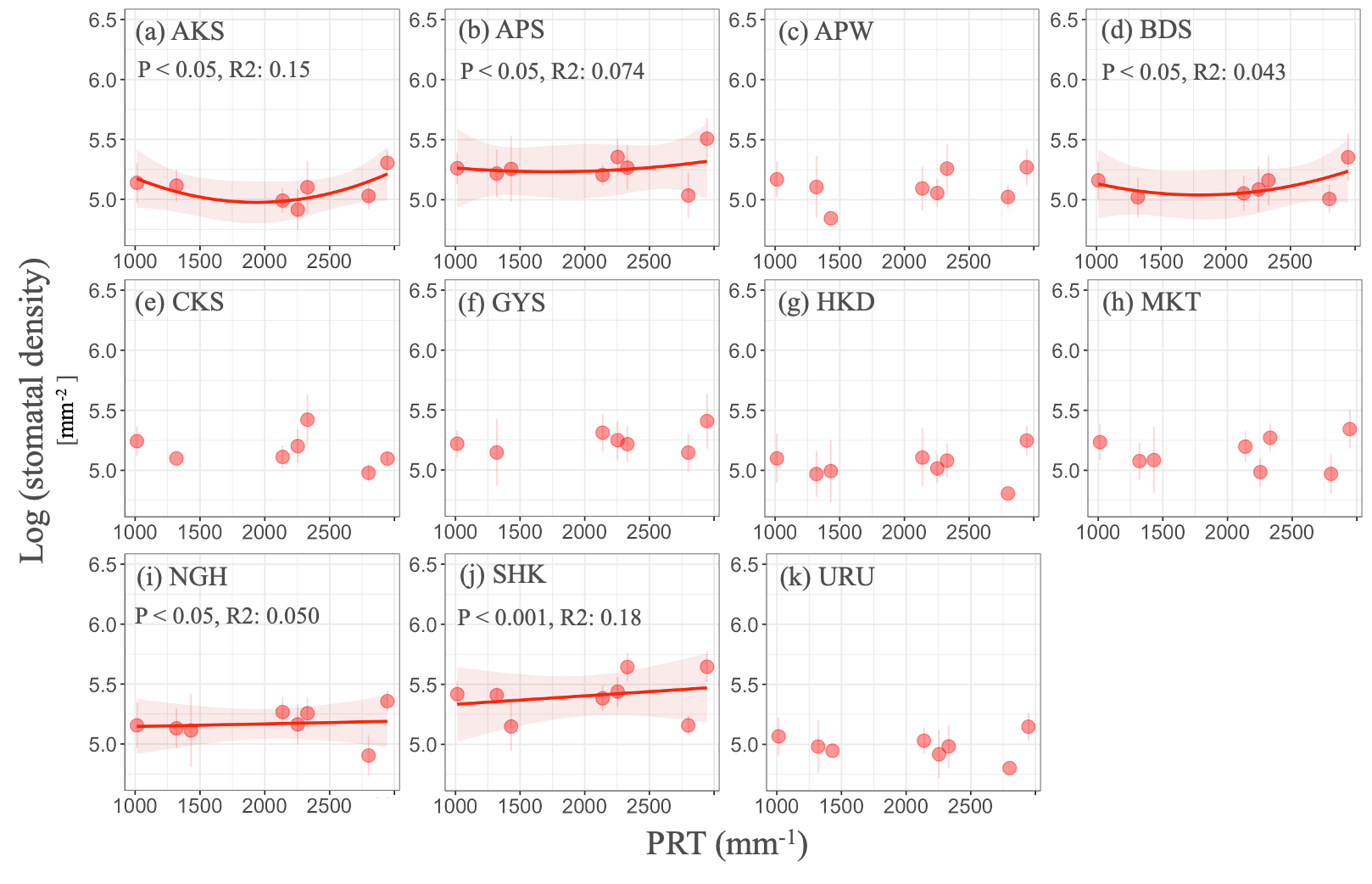


Fig. S6 Relationship between annual precipitation (PRT) of experimental sites and stomatal density of different original sites.
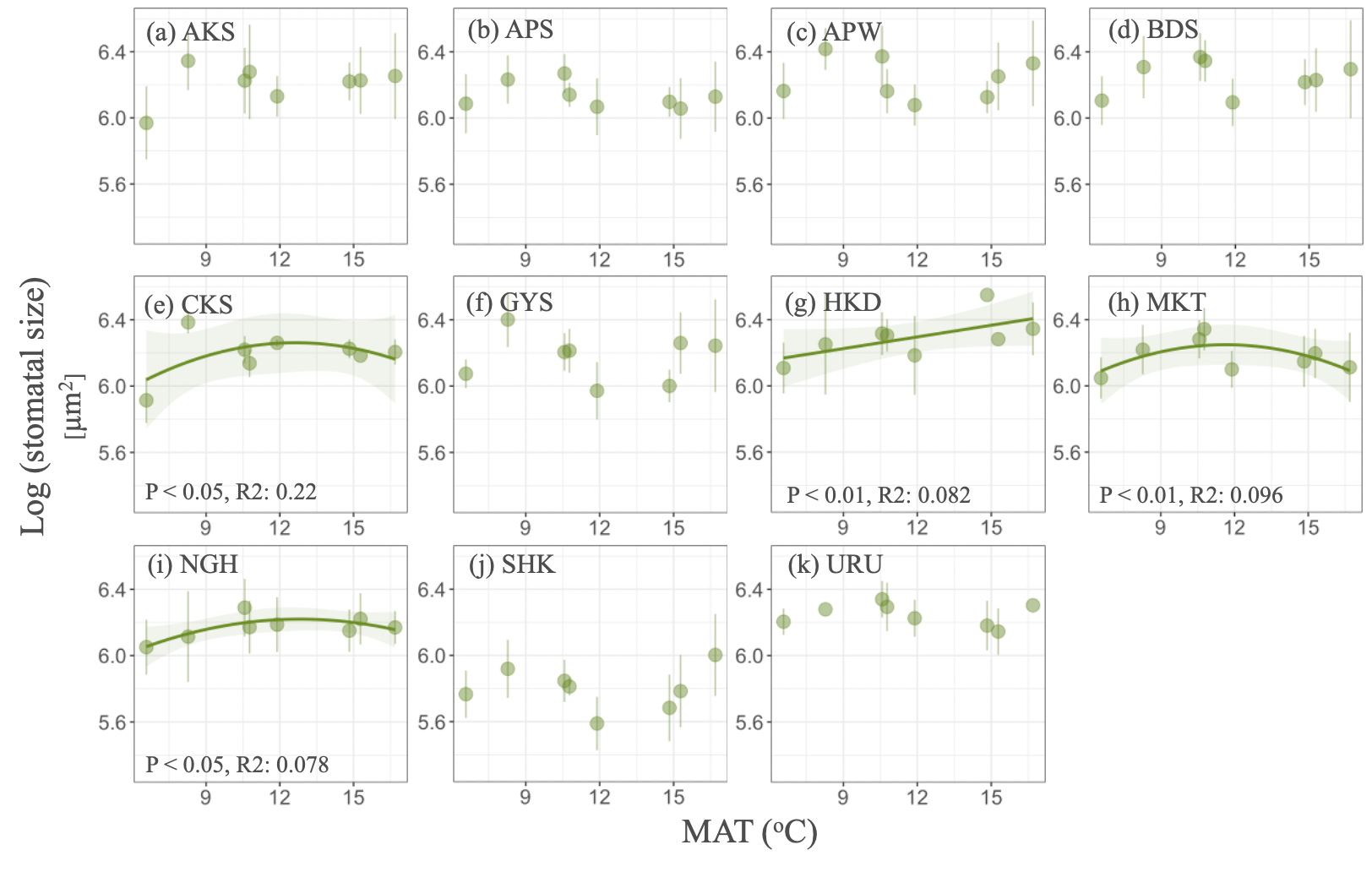


Fig. S7 Relationships between mean annual temperature (MAT) of experimental sites and stomatal size of different original sites.


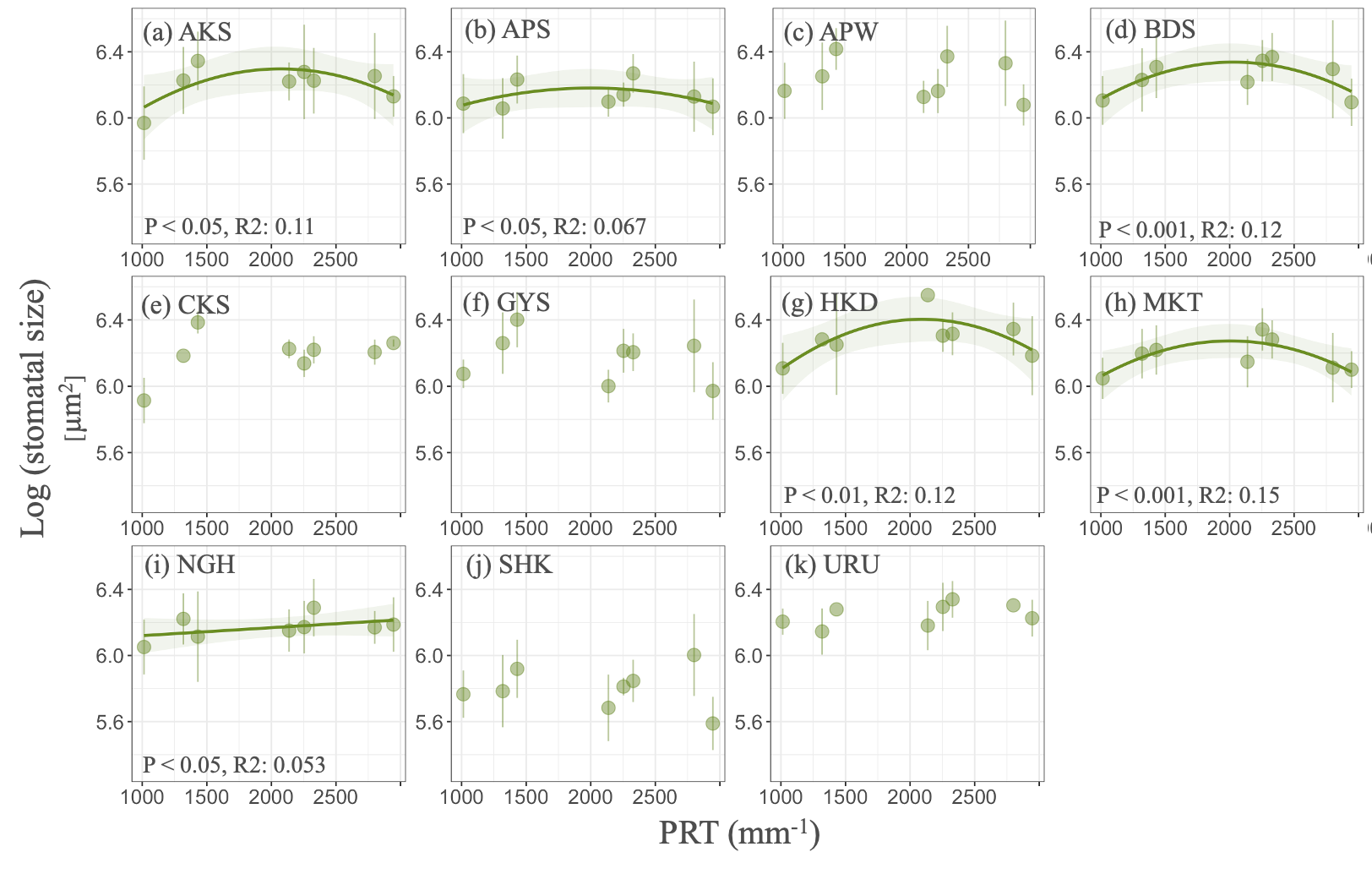


Fig. S8 Relationship between annual precipitation (PRT) of experimental sites and stomatal size of different original sites.

Table S1 Results of ANOVAs of the traits.

|  | df | F value | P value | ratio |
| --- | --- | --- | --- | --- |
| **Stomatal density** |  |  |  |  |
| Experimental site | 7 | 43.81 | <0.001 | 21.34 |
| Original site | 10 | 33.14 | <0.001 | 22.33 |
| Experimental site × Original site | 66 | 1.51 | <0.01 | 2.81 |
| Residuals | 766 | - | - | 53.51 |
| **Stomatal size** |  |  |  |  |
| Experimental site | 7 | 21.97 | <0.001 | 10.42 |
| Original site | 10 | 41.35 | <0.001 | 28.54 |
| Experimental site × Original site | 70 | 1.56 | <0.01 | 3.24 |
| Residuals | 811 | - | - | 57.79 |

df indicate degree of freedom, F indicate Fisher’s F value and the ratio (%) of the variance component of each factor.

Table S2 Results of the principal component analysis (PCA).

|  | PCA factor | |
| --- | --- | --- |
| Variable | 1 | 2 |
| Altitude | 0.50 | -0.15 |
| Mean temperature in summer (TS) | -0.25 | 0.53 |
| Mean annual temperature (MAT) | -0.29 | 0.54 |
| Summer precipitation (PRS) | 0.35 | 0.47 |
| Annual precipitation (PRT) | 0.42 | 0.41 |
| Aridity index (AI) | 0.54 | -0.04 |
| Percentage of variance explained | 53.6 | 38.4 |

The table shows the values of the scores obtained for each independent variable for each factor.
